# The effects of functional training on physical fitness and skill-related performance among basketball players: a systematic review

**DOI:** 10.3389/fphys.2024.1391394

**Published:** 2024-05-09

**Authors:** Shudian Cao, Jia Liu, Zhaoran Wang, Soh Kim Geok

**Affiliations:** ^1^ School of Physical Education, Xihua University, Chengdu, China; ^2^ Department of Physical Education, Yuncheng University, Yuncheng, China; ^3^ School of Physical Education, Qingdao University, Qingdao, China; ^4^ Faculty of Educational Studies, University Putra Malaysia, Putra, China

**Keywords:** functional exercises, speed, power, endurance, agility, balance, dribbling, shooting

## Abstract

**Background:** Evidence suggests that functional training (FT) positively impacts physical fitness and sports performance. However, a systematic review addressing the effects of FT on basketball players remains absent. This systematic review aims to explore the influence of FT on physical fitness and skill-related performance in basketball players.

**Methods:** We searched six databases: Web of Science, Scopus, PubMed, China National Knowledge Infrastructure (CNKI), EBSCOhost, and Google Scholar. The search utilized a combination of keywords related to FT, physical fitness, and basketball. The Eligibility Criteria of Preferred Reporting Items for Systematic Reviews and Meta-Analyses (PRISMA 2020) guidelines were followed in this systematic review.

**Results:** 11 studies were ultimately included in this review, collectively recruiting 333 basketball players. These studies demonstrated that FT significantly improved muscle strength, linear speed, cardiovascular endurance, flexibility, balance, and muscular endurance. However, the effects of FT on power, change-of-direction speed, and basketball-related performance were inconsistent. Most studies showed FT significantly improves these three variables, but a small number of studies did not find positive effects of FT using specific tests including standing long jump, Sargent jump, touch high, lane agility, lateral shuffle, dribbling line drill, and free-throw tests.

**Conclusion:** FT is an effective training method for enhancing physical fitness including muscle strength, linear speed, cardiovascular endurance, flexibility, balance, and muscular endurance. However, the effects of FT on power, change-of-direction speed, and basketball-related performance were divergent. Some tests were not improved after FT potentially due to the short program lengths and training session durations, varied athletic levels of players examined, and different foci of the FT exercises administered. The collective evidence suggests FT programs, especially the specific exercises prescribed, should be tailored to the desired training objectives. More studies investigating the effects of FT on physical fitness and basketball-related performance with established tests are encouraged in the future to expand the current evidence base.

**Systematic Review Registration:** https://inplasy.com/, Identifier INPLASY202360072.

## 1 Introduction

Basketball is an extremely dynamic sport that combines aerobic and anaerobic metabolic contributions (Mancha-Triguero et al., 2020). Consequently, basketball requires well-developed physical fitness and encompasses many specific game activities such as sprinting, jumping, changing direction, accelerations, and decelerations. These activities are performed repeatedly in both offence and defence in basketball (Ramirez-Campillo et al., 2022). Physical training, including power, strength, speed, and balance training, can improve these activities (Cumps et al., 2007; Chaouachi et al., 2009; Dallinga et al., 2012; DiFiori et al., 2018; Kabacinski et al., 2018). For instance, power training like box jumps, medicine ball throws, and explosive push-ups improves the ability of basketball players to make quick, powerful movements such as jumping for a rebound or executing a fast break (Aksovic et al., 2020). Balance training like single-leg squats, balance board drills, and core strengthening exercises enhance the stability and coordination of basketball players, which helps them align their bodies correctly, ensuring a smooth and accurate shot (Boccolini et al., 2013). Coaches and trainers should make the targeted training program for players. In this regard, resistance training has proven to be effective in enhancing physical fitness among athletes (Lesinski et al., 2016) whereby the primary muscle groups are strengthened through lifting or weight-bearing exercises. However, the benefits of strength training cannot be directly transferred to athletic performance (Buchner et al., 1996). Recently, an increasing number of studies have shown that functional training can improve athletic performance in sports. For instance, research has shown that FT programs improve balance in handball players (Elbadry, 2014), power, flexibility, agility, and balance in tennis players (Yildiz et al., 2019), and power and speed in soccer players (Turna and Alp, 2020).

Functional training (FT) can be any type of training that is performed to enhance a certain task or activity. The definition of FT is broad. Boyle (2016) indicated that FT focuses on exercises that mimic the specific movements and demands of a sport or daily activities, such as squat, lunge, shoulder press, deadlift, and high pull exercises. It is a training system designed for acceleration, deceleration, and stability across various joints and dimensions of the body (Boyle, 2004). Unlike other training methods such as small-sided games (SSG) that focus on sport-specific skills and tactical understanding on a smaller field or court (Halouani et al., 2014), or high-intensity interval training (HIIT) that aims to improve cardiovascular fitness and caloric expenditure in a short amount of time (Vasconcelos et al., 2020), FT integrates joints, dynamic tasks, and consistent modifications to train muscles in coordinated and multi-movement patterns (Boyle, 2004). The goal of FT is to improve the abilities of players such as functional strength, agility, balance, and coordination required for optimal performance in sport (Sharrock et al., 2011; Boyle, 2016). FT programs are tailored to the specific movements and physical demands of the sport (Boyle, 2016). For example, the FT program for a basketball player includes exercises that mimic jumping, sprinting, and lateral movements (Usgu et al., 2020). On the other hand, by targeting muscle groups and movement patterns specific to the sport, FT can help reduce the risk of common sports-related injuries. For instance, exercises that strengthen the muscles around the knee can help prevent anterior cruciate ligament (ACL) injuries in basketball players (Fontenay et al., 2013). Based on the collective evidence regarding FT and what it entails, FT in the present review is defined as a form of exercise that emphasizes the development of physical abilities and skills directly applicable to basketball performance and overall physical fitness. It involves multi-joint, multi-plane movements that simulate sports-specific activities, with a focus on enhancing core stability, mobility, strength, power, speed, balance, and coordination. It aims to improve the capacity of players to perform athletic movements more efficiently and with a reduced risk of injury.

Given the definition and characteristics of FT and the nature of basketball, FT emerges as a scientific and professional training approach for basketball players (Kumar, 2014). Specifically, by training muscles to work together in coordinated patterns, FT improves movement efficiency on the court, which leads to better execution of complex movements like pivoting, cutting, and changing direction quickly (Schelling and Torres-Ronda, 2013). On the other hand, FT often includes plyometric and power exercises that mimic the explosive movements in basketball (Santana, 2015; Boyle, 2016), such as jumping for rebounds or blocks and accelerating quickly during fast breaks. The improvement of explosive power enhances the ability of basketball players to generate force rapidly, leading to improved performance in these critical aspects of the game (Attene et al., 2015). In addition, the dynamic nature of FT exercises helps improve balance and stability, which are crucial for maintaining control during shooting, defending, and executing moves (Michell et al., 2006; Curtolo et al., 2017). Better balance also reduces the risk of ankle and knee injuries common in basketball (Taylor et al., 2015).

To the best of our knowledge, several reviews have reported that FT can enhance sport-related performance (Wilke and Mohr, 2020; Xiao et al., 2021; Bashir et al., 2022), but there is a gap in literature specifically investigating the effects of FT on physical fitness and skills of basketball players. Consequently, this systematic review aims to elucidate the impact of FT on physical fitness and skill performance among basketball players.

## 2 Methods

### 2.1 Protocol and Registration

The Eligibility Criteria of Preferred Reporting Items for Systematic Reviews and Meta-Analyses (PRISMA 2020) guidelines were followed in this systematic review (Page et al., 2021). This review was registered on 25 June 2023, on the Platform of Registered Systematic Review and Meta-analysis Protocols (INPLASY202360072).

### 2.2 Eligibility criteria

The following inclusion criteria were set according to the PICOS framework (Table 1): (1) Full-text studies published in English or Chinese; (2) The population consists of healthy basketball players with no limitations on their sexes, age, or level; (3) Studies that used FT, which aligned with the definition adopted in this review, as the intervention in the experimental group; (4) Studies that had control groups not completing a FT program, or studies without control groups; (5) Outcome measures indicative of physical fitness including body composition, muscular endurance, muscular strength, cardiovascular endurance, flexibility, balance, coordination, agility, speed, power, and reaction time (Xiao et al., 2021) or basketball skill-related performance (e.g., shooting or dribbling performance); and (6) Randomized controlled trials (RCTs) or non-randomized controlled trials (nRCTs) with two or more groups, or single-group trials.

**TABLE 1:** Inclusion criteria according to the PICOS condition.

Items	Detailed inclusion criteria
Population	Basketball players across sexes, ages, and levels without injury
Intervention	Functional training
Comparison	Two or more groups and single-group trials
Outcome	Physical fitness or basketball skill-related performance
Study designs	RCTs or nRCTs

*Note*. RCTs, randomized controlled trials; nRCTs, non-randomized controlled trials.

The exclusion criteria were: (1) Reviews; (2) Studies without FT as an intervention; (3) Unpublished studies; and (4) Studies examining wheelchair basketball given the different scope of FT exercises delivered to this population.

### 2.3 Information sources and search strategy

The search was conducted on 3 January 2024. The following databases were used: Web of Science, Scopus, PubMed, China National Knowledge Infrastructure (CNKI), EBSCOhost, and Google Scholar (Table 2). The search terms were “functional training” OR “functional exercise” OR “functional skill*” OR “functional task training” OR “therapeutic exercise” AND basketball. The reference lists within the included studies were also screened.

**TABLE 2:** Number of hits for the complete search strategy of the databases.

Database	Complete search strategy	Hits (3 January 2024)
Web of Science (1991-January 2023)	(TS = (“functional training” OR “functional exercise” OR “functional skill*” OR “functional task training” OR “therapeutic exercise”)) AND TS = (basketball)	25
Scopus (1961-January 2023)	TITLE-ABS-KEY (“functional training” OR “functional exercise” OR “functional skill*" OR “functional task training” OR “therapeutic exercise” AND basketball)	33
PubMed (1977-January 2023)	(“functional training" [Title/Abstract] OR “functional exercise" [Title/Abstract] OR “functional skill*" [Title/Abstract] OR “functional task training" [Title/Abstract] OR “therapeutic exercise" [Title/Abstract]) AND (basketball [Title/Abstract])	14
CNKI	TKA = (“functional training” OR “functional exercise” OR “functional skill*” OR “functional task training” OR “therapeutic exercise”) AND TKA = basketball	22
EBSCOhost (1985-January 2023)	AB (“functional training” OR “functional exercise” OR “functional skill*” OR “functional task training” OR “therapeutic exercise”) AND AB basketball	21
Google Scholar	“Functional training” OR “functional exercise” OR “functional skill*” OR “functional task training” OR “therapeutic exercise” AND basketball	24

### 2.4 Study selection

Endnote software (X20, Thomson Reuters, New York City, NY, United States) was used to remove duplicates. Subsequently, two authors (SC and ZW) independently screened the results based on the title and abstract. Then, two authors (SC and JL) reviewed these studies according to the inclusion criteria and PICOS. All processes were determined through discussion, and any discrepancies (e.g., types of intervention, study design) were resolved with consulting the third author (SKG) if necessary. The Kappa statistic was calculated by SPSS software (IBM Corp. Released 2022. IBM SPSS Statistics for Macintosh, Version 29.0. Armonk, NY: IBM Corp) to determine the agreement between raters throughout the PRISMA process (Narducci et al., 2011).

### 2.5 Data extraction

After selecting the studies, authors (SC and ZW) extracted the data, which included: (1) participant characteristics (sex, age, height, body mass, playing level, and training experience); (2) FT and other interventions; (3) comparison (control group); (4) intervention characteristics (training content, program length, frequency, session duration); (5) assessments (tests used to measure the effect of FT on players); and (8) outcomes (results from pre-to post-intervention and between-group comparisons). Once the information was organized into the Microsoft Excel spreadsheet (XP professional edition; Microsoft, Redmond, WA, United States), another author (SKG) reviewed it for accuracy.

### 2.6 Quality assessment

The 14-item “Qualsyst”, with specific criteria (yes = 2, partial = 1, no = 0), was employed to assess the quality of the studies (Kmet et al., 2004) (Table 3). This assessment tool was used in many reviews with similar topics to the present systematic review (Cao et al., 2022a; Cao et al., 2022b; Bravo et al., 2022). The quality of each included study was assessed independently by two authors (SC and ZW), and any discrepancies were discussed and resolved via consensus with a third author (SKG). This tool categorized the selected studies into strong quality (75% or higher), moderate quality (55%–75%), and poor quality (less than 55%).

**TABLE 3:** Quality assessment through QualSyst.

Studies	I	II	III	IV	V	VI	VII	VIII	IX	X	XI	XII	XIII	XIV	Score	Rating
Hany (2017)	2	2	2	2	0	0	0	2	2	2	2	0	2	2	20	Moderate
Chen (2018)	2	2	2	1	0	0	0	2	2	2	2	0	2	2	19	Moderate
Lukose (2018)	2	2	0	2	2	0	0	2	2	2	0	0	2	2	18	Moderate
Zuo (2018)	2	2	2	2	0	0	0	2	2	2	2	0	2	2	20	Moderate
Usgu et al. (2020)	2	2	2	2	0	0	0	2	2	2	2	0	2	2	20	Moderate
Wibowo et al. (2020)	2	2	2	1	2	0	0	2	2	2	0	0	2	2	19	Moderate
Bhardwaj and Kathayat (2021)	2	2	2	1	0	0	0	2	2	2	2	0	2	2	20	Moderate
Zhang et al. (2021)	2	2	2	1	2	0	0	2	2	2	2	0	2	2	21	Strong
Hovsepian et al. (2021)	2	2	2	1	2	0	0	2	2	2	2	0	2	2	21	Strong
Ding (2022)	2	2	2	1	2	0	0	2	2	2	2	0	2	2	21	Strong
Shang et al. (2023)	2	2	2	2	2	0	0	2	2	2	2	0	2	2	22	Strong

*Note*. Two indicates yes, one indicates partial, 0 indicates no, I question described, II, appropriate study design; III, appropriate subject selection; IV, characteristics described, V random allocation, VI, researchers blinded; VII, subjects blinded; VIII, outcomes measure well defined and robust to bias; IX, sample size appropriate, X analytic methods well described, XI, estimate of variance reported; XII, controlled for confounding; XIII, results reported in detail, and XIV, conclusion supported by results.

### 2.7 Data Synthesis

Meta-analyses of included studies were not able to be conducted given the requirement for comparable outcome measures taken at similar time points (Harrer et al., 2021). In this regard, the included studies did not consistently provide three or more baseline and follow-up measurements for the same variables. Moreover, the included studies did not have sufficient homogeneity regarding the players recruited, interventions administered, and outcome measures taken (Deeks et al., 2019). Consequently, extracted data from the included studies were analyzed according to the Centre for Reviews and Dissemination (Akers et al., 2009).

## 3 Results

### 3.1 Study selection

**FIGURE 1:**
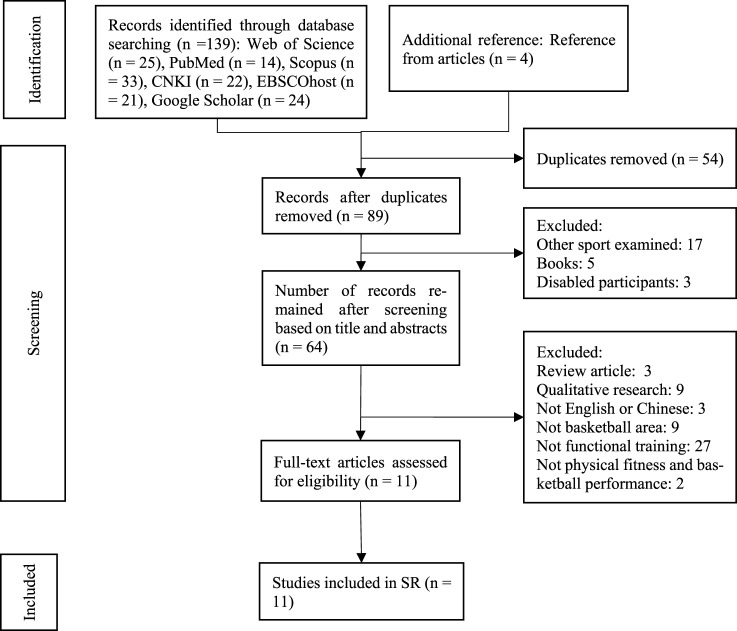
Systematic review search and screening procedure.

We screened a total of 143 studies. After removing duplicates, 89 studies remained. In turn, 64 studies remained for full-text review after titles and abstracts were screened. Then, these studies were assessed according to the inclusion and exclusion criteria. The initial Kappa statistic for agreement between authors was 0.869. Two discrepancies in the screening process were resolved by discussing with the third author. Finally, the agreement Kappa statistic for agreement between authors was 1.00 during full-text screening (Figure 1).

### 3.2 Study quality assessment

Two authors independently assessed the quality of the 11 studies according to the “Qualsyst”, and the Kappa statistic was 0.876. Four of the 11 selected studies were of high quality (Hovsepian et al., 2021; Zhang et al., 2021; Ding, 2022; Shang et al., 2023). The remaining seven studies were of moderate quality (Hany, 2017; Chen, 2018; Lukose, 2018; Zuo, 2018; Usgu et al., 2020; Wibowo et al., 2020; Bhardwaj and Kathayat, 2021). No studies were excluded based on their quality.

### 3.3 Participant characteristics

The population characteristics of the 11 studies were reported based on the following:

(1) Sample size. Across all studies, 333 participants were included, ranging in sample sizes from 10 (Hany, 2017) to 80 (Zhang et al., 2021) participants, with a mean sample size of 30 participants (SD = 22).
(2) Sex. Six studies investigated males (Hany, 2017; Lukose, 2018; Usgu et al., 2020; Wibowo et al., 2020; Bhardwaj and Kathayat, 2021; Zhang et al., 2021), one study investigated females (Hovsepian et al., 2021), with four studies not reporting the sex of players (Chen, 2018; Zuo, 2018; Ding, 2022; Shang et al., 2023);
(3) Level. Four studies investigated professional basketball players (Hany, 2017; Usgu et al., 2020; Ding, 2022; Shang et al., 2023), four studies investigated collegiate basketball students (Chen, 2018; Zhang et al., 2021; Ding, 2022; Shang et al., 2023), with three studies not reporting the level for players (Lukose, 2018; Zuo, 2018; Bhardwaj and Kathayat, 2021).

### 3.4 Intervention characteristics

The characteristics of the included studies were as follows:

(1) Training program length: The training program length ranged from 4 weeks (Chen, 2018) to 20 weeks, and the mean training program length was 10.5 weeks (SD = 4.8) (Usgu et al., 2020).
(2) Training duration: Only two studies specified the training duration of the intervention, which were 21 min (Wibowo et al., 2020) and 30 min (Ding, 2022) per session. The remaining nine studies did not provide this detail (Hany, 2017; Chen, 2018; Lukose, 2018; Zuo, 2018; Usgu et al., 2020; Bhardwaj and Kathayat, 2021; Hovsepian et al., 2021; Zhang et al., 2021; Shang et al., 2023).
(3) Training frequency: Seven studies detailed the training frequency of the intervention (Hany, 2017; Usgu et al., 2020; Wibowo et al., 2020; Bhardwaj and Kathayat, 2021; Hovsepian et al., 2021; Ding, 2022; Shang et al., 2023), which varied from 2 to 4 times per week. The other four studies did not specify the frequency (Chen, 2018; Lukose, 2018; Zuo, 2018; Zhang et al., 2021).
(4) The definition of FT in included studies is shown in Table 4. These definitions meet the inclusion criteria for intervention in the present systematic review.

**TABLE 4:** Definition of functional training in included studies.

Study	Definition in each study
Hany (2017)	FT allows one exercise to work out a much larger number of muscles to develop not only strength but also still dexterity and flexibility. The sand in the Bulgarian bag is necessary to use more force, energy, muscle groups and physical reserves of the body than when training with “iron” of the same weight in traditional resistance training
Chen (2018)	FT emphasizes the overall concept of training, emphasizes the value of core area strength, and emphasizes the multi-directional and multi joint linkage during the exercise process
Lukose (2018)	FT focuses on strengthening core strength to improve sports performance
Zuo (2018)	FT aims to improve overall physical fitness including explosive power, strength, and agility
Usgu et al. (2020)	FT attempts to train muscles in coordinated, multiple-movement patterns and incorporates joints, dynamic tasks, and consistent alterations for functional improvement. To improve performance, exercise training in FT should be performed in specific movement patterns required by different sports
Wibowo et al. (2020)	FT is an exercise that can activate several muscular groups, especially the core or core muscles. The difference between FT with other training such as traditional weight training lies in the tools, muscle focus, training methods, and training objectives
Bhardwaj and Kathayat (2021)	FT is to accurately perform fundamental movement patterns and maintain an optimal balance between mobility and stability
Zhang et al. (2021)	FT is a synthesis of training actions that aim to improve balance, stability, core strength, and dynamic motion abilities
Hovsepian et al. (2021)	High-intensive FT is a series of exercises that focus on high intensity, functionality, variability, and flexibility
Ding (2022)	FT emphasizes the movement chain of the body, efficient movement patterns, and basic flexibility and stability during the training process
Shang et al. (2023)	FT focuses on building the construction of motion models and a solid foundation in terms of physical stability, physical strength, and more

### 3.5 Outcome characteristics

#### 3.5.1 Effect of FT on Power

Seven selected studies examined the impact of FT on power. The power Table 4 measured in selected studies could be divided into upper limb power, lower limb power, and anaerobic power. The assessment tools used to measure lower limb power included the Sargent jump (Hany, 2017; Hovsepian et al., 2021), countermovement jump (Usgu et al., 2020), jump with the basketball (Chen, 2018), standing long jump (Zuo, 2018; Usgu et al., 2020; Zhang et al., 2021), touch high (Shang et al., 2023), and run-up touch high (Shang et al., 2023). The assessment tool used to measure upper limb power was medicine ball throw (Hany, 2017). The assessment tool used to measure anaerobic power was the running-based anaerobic sprint test (RAST) (Hovsepian et al., 2021). Some studies noted significant improvements in the Sargent jump (Hany, 2017), countermovement jump (Usgu et al., 2020), throwing the medicine ball (Hany, 2017), jump with the ball (Chen, 2018), standing long jump (Zuo, 2018), RAST (Hovsepian et al., 2021) and run-up touch high (Shang et al., 2023) after FT. However, some studies indicated no significant effects with FT in the standing long jump (Usgu et al., 2020; Zhang et al., 2021), Sargent jump (Hovsepian et al., 2021), and touch high (Shang et al., 2023) tests.

#### 3.5.2 Effect of FT on Muscle Strength

Five studies examined the impact of FT on strength, using assessment tools such as pull-up (Hany, 2017; Zuo, 2018; Zhang et al., 2021), leg press (Usgu et al., 2020), bench press (Usgu et al., 2020), and push-up (Shang et al., 2023) tests. The studies indicated that FT significantly improved performance in all of these tests.

#### 3.5.3 Effect of FT on Speed

According to the assessments used in selected studies, speed was divided into change of direction (COD) speed and linear speed.

Seven studies reported on the impact of FT on COD speed, using assessment tools such as the T-test (Zuo, 2018; Usgu et al., 2020; Bhardwaj and Kathayat, 2021; Hovsepian et al., 2021), lane agility test (Usgu et al., 2020), lateral shuffle test (Hovsepian et al., 2021), side-step test (Wibowo et al., 2020), triangle side slide (Ding, 2022), and 17 turns back (Shang et al., 2023). FT significantly improved performance in most of these tests except for the lane agility (Usgu et al., 2020) and lateral shuffle tests (Hovsepian et al., 2021). Four studies showed that FT could significantly enhance linear speed including 20-m sprint (Hany, 2017; Usgu et al., 2020), 40-m sprint (Hany, 2017), and 50-m sprint (Bhardwaj and Kathayat, 2021; Zhang et al., 2021) tests.

#### 3.5.4 Effect of FT on Cardiovascular Endurance

Four studies suggested that FT could significantly enhance cardiovascular endurance in the 12-min run (Zuo, 2018), 1000-m run (Zhang et al., 2021), basketball-specific field test (Hovsepian et al., 2021), and 3200-m run (Shang et al., 2023).

#### 3.5.5 Effect of FT on Flexibility

Three studies indicated that FT could significantly enhance flexibility performance in the sit and reach (Usgu et al., 2020; Zhang et al., 2021) and body acuity detection (Shang et al., 2023) tests.

#### 3.5.6 Effect of FT on Balance

Two studies demonstrated that FT could significantly enhance balance performance in standing on one leg with eyes closed (Zuo, 2018) and the balance beam test (Wibowo et al., 2020) tests.

#### 3.5.7 Effect of FT on Muscular Endurance

Only one study showed a significant improvement on muscular endurance with FT using the plank test (Zuo, 2018).

#### 3.5.8 Effect of FT on Basketball-related Skills

Five studies examined the impact of FT on basketball-related skills, using tests including the footwork and hook shot (Hany, 2017), dribble obstacle (Chen, 2018), 1-min shot (Chen, 2018; Zhang et al., 2021), dribbling line drill (Chen, 2018), free-throw (Chen, 2018), shooting (Lukose, 2018), “V” layup (Zhang et al., 2021), 30-s quick shot (Ding, 2022), and layup after dribbling (Ding, 2022) tests. However, FT had no effect on performance during the dribbling line drill and free-throw tests (Chen, 2018).

## 4 Discussion

The aim of this systematic review was to elucidate the effects of FT on physical fitness and skill-related performance in basketball players. The findings suggest that FT can significantly enhance muscle strength, linear speed, cardiovascular endurance, flexibility, balance, and muscular endurance of basketball players. FT can also improve power, COD speed, and basketball-related performance, but there were exceptions in certain tests (standing long jump, Sargent jump, touch high, lane agility, lateral shuffle test, dribbling line drill, and free-throw). Notably, no data were available regarding the impact of FT on body composition, agility, reaction time, and coordination.

### 4.1 Effect of FT on Power

Power in basketball is a multifaceted attribute that influences the performance of players in numerous ways. Powerful players can be more imposing defensively, challenging shots, guarding multiple positions, and providing help defence (Aksovic et al., 2021). The results showed that FT had a significant improvement in upper limb power (throwing the medicine ball) (Hany, 2017) and anaerobic power (running-based anaerobic sprint test) (Hovsepian et al., 2021). Upper limb power is pivotal in basketball for actions like passing, dribbling, and defence (Cabarkapa et al., 2022). Anaerobic power refers to the ability of an athlete to exert maximum effort in short bursts of high-intensity activity, which is crucial in basketball due to its fast-paced and explosive nature (Stauffer et al., 2010). However, given only one study examined each of upper limb power and anaerobic power, the evidence is not comprehensive to date, so more studies are needed examining the effects of FT on these forms of power in the future.

On the other hand, all the studies selected for this systematic review utilized jump-related tests to measure lower limb power, primarily including the vertical jump (countermovement jump, Sargent jump), horizontal jump (standing long jump), and run-up vertical jump. Basketball is typified by explosive power and unilateral actions, such as jumping (Fort-Vanmeerhaeghe et al., 2016; Makaruk et al., 2020). In basketball, vertical jumps are crucial for executing key technical actions like shooting, blocking, and rebounding (Aksovic et al., 2022). Based on the results from the selected studies, the impact of FT on lower limb power appears to be contentious, aligning with previous systematic evidence (Xiao et al., 2021). Lower limb power depends on several factors such as muscle strength and neuromuscular coordination (Hammami et al., 2019). Some plyometric training including box jumps and depth jumps that was used in the FT programs among the included studies (Zuo, 2018) is highly beneficial for improving neuromuscular coordination, and the squats and hip bridge training (Usgu et al., 2020) could improve lower-limb strength. Therefore, these studies showed a significant improvement in lower limb power after FT. Usgu et al. (2020) and Zhang et al. (2021) showed the FT did not have a significant effect on the standing long jump, which might be because basketball players are more accustomed to vertical jumps than horizontal jumps given the execution of fundamental tasks such as rebounding, shooting, and blocking shots in training and games. Moreover, the lack of effects for FT on Sargent jump performance reported by Hovsepian et al. (2021) may be explained by the nature of Sargent jump. Sargent jump typically involves a static start without a preceding downward movement, which limits the use of the stretch-shortening cycle (SSC). FT program in studies often included a variety of jump tasks that engage the SSC. If the FT focused more on jump tasks involving SSC, the training might not have adequately targeted the specific muscular and neuromuscular adaptations required to improve performance in the Sargent jump test. On the other hand, the recruitment of female players in this study (Hovsepian et al., 2021) might be another reason, given women generally have less muscle mass and different hormone profiles compared to men, which can influence how they respond to strength and power training (Buchanan and Vardaxis, 2009).

### 4.2 Effect of FT on Change of Direction (COD) Speed

COD speed is a critical skill in basketball that significantly impacts the performance of players on the court. It involves the ability to quickly and efficiently alter direction while maintaining control and balance (Scanlan et al., 2015; Taylor et al., 2017; Stojanović et al., 2018). The results of FT on COD speed were contentious, which is similar to a previous systematic review encompassing many sports (Xiao et al., 2021). Most of the included studies reported a significant improvement of FT on COD speed in assessments like the T-test (Zuo, 2018; Usgu et al., 2020; Bhardwaj and Kathayat, 2021; Hovsepian et al., 2021), side-step test (Wibowo et al., 2020) and turned back test (Shang et al., 2023). The improvement of COD speed may be due to the FT program (Table 5) in these studies. For instance, the BOSU V-sit ups, unilateral leg-raising, and hip rotation can improve core strength, mobility and stability, which are essential for maintaining balance and control during quick changes in direction (Czyżnielewska et al., 2023). Likewise, the improvements in COD speed with FT could be due to the interaction of neuromuscular adaptations. Specifically, functional exercises require coordination between multiple muscle groups and the nervous system (Boyle, 2016). As athletes become more adept at these movements, their neuromuscular coordination improves, allowing for more efficient and controlled changes in direction (Arede et al., 2022). In addition, FT challenges balance and proprioception (the sense of position and movement in space). Improved proprioception helps athletes maintain stability and control during rapid directional changes (Ergen and Ulkar, 2007; Šalaj et al., 2007). However, two studies indicated the FT did not significantly improve performance in the lane agility (Usgu et al., 2020) and lateral shuffle test (Hovsepian et al., 2021). A reason for the non-significant findings in these studies might relate to the professional level of the players examined. As professional athletes, their training history is extensive and varied, which means their bodies have adapted to numerous stimuli over the years (Cormie et al., 2010). Accordingly, the FT might not have provided sufficient stimuli to elicit significant improvements in these specific COD speed tests. Given the varying results, more research on this topic is encouraged to gather a definitive understanding.

**TABLE 5:** Overview of FT on physical fitness and sport-related performance in basketball players.

Study	Population characteristic	I	Comparison	Intervention characteristic		Assessments	Outcome	
				Train content	Length/Freq/Dura		Pre-post	Groups
Hany (2017)	N: 10 M; A: 20.67 ± 1.9 years; H: 198 ± 8.7 cm; BM: 92 ± 7.3 kg; L: Professional players	FT	N/A	Functional exercises with Bulgarian bag	Freq: 4 sessions/week; Length: 8 weeks	Power (SJ, throwing the medicine ball); muscle strength (chin up); linear speed (20-m, 40-m sprint); BS (pivot footwork and hook shot)	All↑	N/A
Chen (2018)	N: 16; A: NR; H: NR; BM: NR; L: Collegiate players	FT	Traditional physical training	Training with Balance board, Swiss ball	Length: 4 weeks	BS (dribble obstacle, 1-min shooting, dribbling line drill, free-throw); power (jump with the basketball)	EG: dribble obstacle, 1-min shot, jump with the basketball↑; dribbling line drill, free-throw ↔	Dribble Obstacle, 1-min shot, jump with the basketball ↑ in, EG vs. CG; dribbling line drill, free-throw ↔ in, EG vs. CG
							CG: all ↔	
Lukose (2018)	N: 45 M; A: 18–25 years; H: NR; BM: NR; L: NR	EG1: FT	Without any experimental training	NR	Length: 12 weeks	Shooting	EG1: Shooting ↑	Shooting ↑in, EG1 and, EG2 vs. CG; Shooting ↔ in, EG2 vs. EG1
		EG2: plyometric training					EG2: Shooting ↑	
							CG: Shooting ↔	
Zuo (2018)	N: 12; A: 18.50 ± 1.4 years; H: 181.1 ± 3.5 cm; BM: 65.9 ± 5.7 kg; L: NR	FT	N/A	Box Jump; Depth Jump; bench press; squat	Length: 14 weeks	Cardiovascular endurance (12-min run); balance (stand up on one leg with eyes closed); muscle strength (pull up); muscular endurance (plank); power (SLJ), COD speed (T-test)	All↑	N/A
Usgu et al. (2020)	N: 18 M; A: 25.5 ± 5.0 years; H: 198 ± 9.3 cm; BM: NR; L: Professional players	FT	Traditional strength training	Mat/Swiss ball; Push-Up; Jack Knife; Hip bridge; Russian Twist; Planks	Freq: 2 sessions/week; Length: 20 weeks	Muscle strength (bench press, leg press); flexibility (sit and reach); COD speed (T-test, lane-agility); linear speed (20-m spring); power (CMJ, SLJ)	EG: all ↑ except lane-agility and SLJ↔	All ↔ except T-test, Lane-agility, and leg press↑ in, EG vs. CG
							CG: all ↑ except sit and reach, SLJ, CMJ, and T-test ↔	
Wibowo et al. (2020)	N: 24 M; A: 13–15 years; H: NR; BM: NR; L: Professional players	FT	Usually exercises	Circuit training using the AMRAP: BOSU V-sit ups; VIPR side balance; TRX single leg balance	Freq: 3 sessions (21 min)/week; length: 6 weeks	Balance (balance beam test); COD speed (side-step test)	EG: all↑	All ↑ in, EG vs. CG
							CG: NR	
Bhardwaj and Kathayat (2021)	N: 20 M; A: 18–24 years; H: NR; BM: NR; L: NR	FT	N/A	Deep Squat; Hurdle step, Active Straight Leg Raise; Trunk Stability push up; Balance and Coordination Exercise followed by foam rolling in cool down procedure	Freq: 2 sessions/week; Length: 6 weeks	Linear speed (50-m sprint); COD speed (T-test)	All↑	N/A
Zhang et al. (2021)	N: 80 M; A: NR; H: NR; BM: NR; L: Collegiate players	FT	Traditional physical training	Upper and lower limb strength; Upper limb + core stability; lower limb + core rotation; hip extensor group	Length: 16 weeks	Linear speed (50-m sprint); cardiovascular endurance (1000-m run); muscle strength (pull-up); power (SLJ); flexibility (sit and reach); BP (“V” layup, 1-min shot)	EG: All ↑ except SLJ ↔	All ↑ in, EG vs. CG
							CG: pull-up, sit and reach, 1-min shot ↑; others ↔	
Hovsepian et al. (2021)	N: 20 FM; A: 222.2 ± 2.5 years; H: 172.0 ± 6.0 cm; BM: 65.0 ± 5.2 kg; L: Professional players	High-intensive FT	Common strength and conditioning training	Different combinations of weightlifting, gymnastics and metabolic conditioning	Freq: 4 sessions/week; Length: 10 weeks	cardiovascular endurance (VO_2_max in BSFT); power (RAST, SJ); COD speed (T-test, LST)	EG: VO_2_max, t-test, RAST, ↑; SJ, LST↔	RAST ↑ and others ↔ in, EG vs. CG
							CG: VO_2_max, t-test ↑, others ↔	
Ding (2022)	N: 60 M; A: NR; H: NR; BM: NR; L: Collegiate players	FT	Traditional physical training	Split step; side-bridge; skip-step; unilateral leg-raising; hip rotation	Freq: 3 sessions (30 min)/week; Length: 12 weeks	COD speed (triangle side slide); BS (30-s quick shot, layup after dribbling)	EG: All ↑	All ↑ in, EG vs. CG
							CG: All ↑	
Shang et al. (2023)	N: 18 M; A: NR; H: NR; BM: NR; L: Collegiate players	FT	Conventional physical training	NR	Freq: 3 sessions/week; Length: 8 weeks	Muscle strength (push-up); cardiovascular endurance (3200-m run) power (touch high, run-up touch high); flexibility (body acuity detection); COD speed (17 turns back)	EG: All ↑ except touch high ↔	Body acuity detection, 17 turns back, run-up touch high ↑; push-up, touch high, 3200-m run ↔ in, EG vs. CG
							CG: All ↑ except touch high, body acuity detection ↔	

*Note*. A, age; C, control; FM, female; M, male; H, height; BM, body mass; TE, training experience; L: level; I, intervention; NR, not reported, N/A, not applicable; FT, functional training; CG, control group; EG, experimental group; BEST, basketball exercise simulated test; RAST, Running-Based Anaerobic Sprint Test; BSFT, basketball-specific field test; VJ, vertical jump; BS, basketball skills; COD, change of direction; HJ, horizontal jump; LST, lateral shuffle test; CMJ, countermovement jump; SJ, sargent jump; VIPR, vitality, performance, and reconditioning; TRX, total resistance exercises; ↑, significantly positive effect; ↔, no effect.

### 4.3 Effect of FT on Linear Speed

Linear speed is an important attribute in basketball, such as in fast breaks, transition defence, and during off-ball movement (Taylor et al., 2017; Stojanović et al., 2018). The results illustrated the significant improvement in linear sprints across 20–40 m in basketball players with FT. These results are not in line with those reported in a previous systematic review (Bashir et al., 2022) examining athletes from different team sports. Bashir et al. (2022) reported that the improvement in some linear speed performance among athletes after FT was not observed in a small number of studies due to the short duration and frequency of the training sessions, as well as the absence of additional exercises accompanying the FT interventions. However, the studies included in our review may have incorporated FT stimuli that enhanced the coordination between the nervous system and muscles, which is important for executing the complex movements involved in sprinting (Keiner et al., 2022). Better coordination can lead to more efficient movement patterns and faster speeds (Wang et al., 2022). On the other hand, some exercises included in the FT program such as jump, squat, plyometrics, and explosive lifts could build strength and improve power output in muscles, which are crucial for quick starts and rapid acceleration (Cronin and Hansen, 2005; Nimphius et al., 2010).

### 4.4 Effect of FT on Muscle Strength

Strength training is a foundational component for the physical conditioning of basketball players, enabling them to move more swiftly, increase power, and reduce injury risk (Wang et al., 2006). The results showed a significant improvement in upper limb (pull-up, push-up, bench press) and lower limb (leg press) muscle strength after FT. This improvement depends on several factors. The compound exercises in the FT programs among the included studies such as squats (Zuo, 2018), push-ups (Usgu et al., 2020), and Bulgarian bag exercises (Hany, 2017) work several muscle groups simultaneously, which are more effective in building overall strength compared to isolation exercises. The plyometrics in FT programs such as depth jumps and box jumps (Zuo, 2018) help develop the fast-twitch muscle fibres, which are responsible for producing power and strength during quick, intense movements (Gervasi et al., 2018). Overall, given only a few studies examined the effects of FT on muscular strength, it is difficult to draw definitive conclusions with further investigations needed to confirm these initial findings.

### 4.5 Effect of FT on Cardiovascular Endurance

Cardiovascular endurance is paramount in basketball. A player who competes throughout all four quarters without substitution might cover a distance ranging from 5,000 m to 6,000 m, with 15%–20% at a moderate pace and 5% at high to maximum speeds (Klusemann et al., 2013). Robust cardiovascular endurance can sustain these intense activities throughout the game. Four studies indicated that FT could enhance performance in the 12-min run, 1000-m run, 3200-m run, and basketball-specific field test (Zuo, 2018; Hovsepian et al., 2021; Zhang et al., 2021; Shang et al., 2023). The high-intensity nature of the FT used in the included studies can elevate heart rate and challenge the cardiovascular system to improve cardiovascular endurance (Ben-Zeev and Okun, 2021). On the other hand, the use of multiple muscle groups and complex movements in FT heavily stress aerobic metabolic pathways (Cress et al., 1996). This increased demand on the cardiovascular system can lead to improved endurance and VO_2_ max over time. Finally, FT improves movement patterns and biomechanics (Carr et al., 2002; Garbenytė-Apolinskienė et al., 2018), which can lead to more efficient use of energy during aerobic activities. Better movement efficiency reduces unnecessary energy expenditure, allowing for improved endurance performance (Morris et al., 2019; Willis et al., 2019; Mangona et al., 2024).

### 4.6 Effect of FT on Flexibility

Flexibility allows for a greater range of motion in the joints, which is essential for executing various basketball skills, such as shooting, dribbling, and rebounding. A greater range of motion can lead to more efficient and effective movements on the court (Woolstenhulme et al., 2006; Notarnicola et al., 2017). Good flexibility also can help reduce the risk of injuries (Cejudo, 2021). The results showed a significant improvement in flexibility after FT. Two studies did not detail the FT program implemented (Lukose, 2018; Shang et al., 2023), making it difficult to explain how the intervention might have improved flexibility. However, some general aspects applied in FT might help explain this improvement. First, FT often includes dynamic exercises that mimic sports movements. These movements require the body to stretch and move through different planes of motion (Boyle, 2016), which can gradually increase flexibility. In addition, some FT routines include foam rolling or other myofascial release techniques (Lee et al., 2022). These techniques can help to release tightness in the muscles and fascia (Paolini, 2009; Manheim, 2017), improving flexibility and range of motion. Therefore, the details of FT intervention are important. When researchers clearly detail the FT program, including exercises, intensity, duration, and frequency, it allows other readers or trainers to replicate the study to verify findings, explore the efficacy of the program further, or compare it against other interventions. Without this clarity, replicability is compromised, limiting the utility and credibility of studies. Further investigations should clearly indicate the FT program adopted for readers to understand the intervention and how it may be effective or not.

### 4.7 Effect of FT on Balance

Maintaining good balance provides a stable, upright, and consistent foundation, which is essential across basketball activities including running, defending, shooting, dribbling, passing, and rebounding (Boccolini et al., 2013). Two studies reported that FT enhanced performance in standing on one leg with eyes closed and the balance beam test (Zuo, 2018; Wibowo et al., 2020). The training used in the FT program could explain this improvement. For instance, the box jump involves jumping onto and off a box or platform. It requires coordination, power, and stability, especially when landing (Sabillah et al., 2022). Regularly performing box jumps can enhance proprioception, lower body strength, and the ability to control the body during dynamic movements (Saputra, 2019), all of which are important for maintaining balance. Likewise, depth jumps involve stepping off a box and immediately jumping vertically upon landing (Clutch et al., 1983). This exercise challenges the ability of the body to absorb impact and quickly generate force (McClenton et al., 2008), which can improve neuromuscular control and stability. These adaptations are beneficial for maintaining balance on unstable surfaces or when changing directions quickly. VIPR (vitality, performance, and reconditioning) side balance exercise involves holding a VIPR (a weighted, tube-shaped tool) and performing various movements that challenge balance and stability. By moving the VIPR to different positions, such as overhead or to the side, the centre of gravity shifts, requiring the body to adjust and maintain balance (Wibowo et al., 2020). TRX (total resistance exercises) single-leg balance exercise uses the TRX suspension trainer, involving standing on one leg while holding onto the TRX straps for support (Aslani et al., 2018). The instability of the suspension system challenges the body to maintain balance, engaging the core, hip stabilizers, and ankle muscles (Abtahi et al., 2023). This exercise is particularly effective for improving unilateral balance (Semprini, 2018; Rausch, 2020), which is directly related to tests like standing on one leg with eyes closed.

### 4.8 Effect of FT on Muscular Endurance

Muscular endurance is the ability of a muscle, or a group of muscles, to keep working against resistance. Muscular endurance allows players to maintain a high level of performance throughout the game, which is essential given the duration and intensity of a basketball game (Singh and Kaur, 2019; Serin and Mehmet, 2021). Zuo (2018) employed the plank as an assessment tool, demonstrating that FT bolstered muscular endurance (Zuo, 2018). Trainers often utilize the plank to develop the core strength of players. A strong core mitigates or prevents injuries during basketball games but also aids players in maintaining control in intense competitions (Sannicandro and Cofano, 2017). However, it is difficult to explain the mechanism of how FT improved muscular endurance in detail because of the limited evidence. Therefore, more research is needed in the future to make an authoritative conclusion about the effect of FT on the muscular endurance of basketball players.

### 4.9 Effect of FT on Basketball Skill-related Performance

With the significant improvement of physical fitness, results showed that FT also significantly improved basketball performance, including shooting performance (pivot footwork and hook shot, 1-min shot, 30-s shot), dribbling performance (dribble obstacle), and layup performance (“V” layup, layup after dribbling).

The improvement of skill-related performance could be from several aspects. First, FT exercises that target the core, such as planks and medicine ball throws that are used in included studies (Zuo, 2018; Usgu et al., 2020), can enhance the stability and power transfer from the lower body to the upper body during the shooting motion (Aksovic et al., 2020). On the other hand, the upper and lower body power improved by FT are important to basketball skill-related performance (Aksovic et al., 2020; Cabarkapa et al., 2022). Functional exercises like push-ups, pull-ups, and dumbbell presses can help build the necessary upper body strength to shoot the ball with force and accuracy over longer distances. The power for a jump shot or a free-throw primarily comes from the legs (Čabarkapa et al., 2020). FT exercises like squats, lunges, and plyometric drills (e.g., box jumps, and squat jumps) can improve lower body strength and power, leading to a more explosive and effective shooting motion. Regarding the improvement of dribbling performance, the core strength increased by FT may contribute to execution with this activity (Luo et al., 2023). A strong and stable core is essential for maintaining balance and control while dribbling, especially when under defensive pressure (Moselhy, 2020). FT exercises that strengthen the core, such as planks and core rotations, can help maintain a solid foundation during dribbling manoeuvres (Feng et al., 2024). FT often includes exercises like single-leg exercises or balance board drills that challenge balance and proprioception (Nikolaos et al., 2012; Zacharakis et al., 2020). Improved proprioception can help players maintain control of the ball and their body position while navigating through defenders. Finally, successful layups often require adjusting the body position in mid-air to avoid defenders or alter the angle of the shot. FT that includes balance exercises and proprioceptive drills can improve body awareness and control, allowing players to make these adjustments effectively (Zacharakis et al., 2020).

However, one study reported that dribbling line drill and free-throw performance were not improved after FT (Chen, 2018). The short training program length (4 weeks) compared to other studies (8–16 weeks) might be the reason. The body may require more than 4 weeks to adapt to new training stimuli. This adaptation includes neurological adaptations, muscle coordination, and energy system development, which might not be fully developed in such a short time frame.

## 5 Limitations

While this study offers significant evidence regarding the benefits of FT on the physical fitness and skill-related performance of basketball players, several limitations should be acknowledged. Firstly, only one study focused on female participants, and six studies did not specify the sex of the participants (Hovsepian et al., 2021). This omission could influence the results, given the distinct differences in physical fitness between males and females (Altavilla et al., 2017). Furthermore, two studies did not provide details of the FT program (Lukose, 2018; Shang et al., 2023), and some specific basketball skill-related tests in studies were not clear. For instance, two studies did not respectively provide how to measure the free-throw (Chen, 2018) and shooting (Lukose, 2018) in the test. The incomplete information might hinder a comprehensive analysis. In addition, the absence of a control group in three studies (Hany, 2017; Zuo, 2018; Bhardwaj and Kathayat, 2021) may introduce bias regarding the true effects of the intervention. Finally, while this review adopted a specific operational definition of FT to guide the inclusion criteria and analysis, it is acknowledged that the concept of FT encompasses a broad spectrum of training methodologies and activities. This inherent diversity within the field of FT is reflected in the wide range of training approaches observed across the included studies. Although this variability might impact the interpretation of the specific effects and benefits of FT, it also underscores the multifaceted nature of FT as a concept that is adaptable to various physical fitness and sports performance goals.

## 6 Conclusion

This systematic review, encompassing eleven published studies, provides compelling evidence that FT can enhance both physical fitness and skill-related performance in basketball players. Specifically, FT has been shown to improve linear speed, cardiovascular endurance, balance, muscular endurance, muscular strength, and flexibility. While most studies highlighted the positive impacts of FT on power, COD speed, and basketball-specific skills performance in some tests, certain performances did not see significant improvements. Factors such as short program length and training session durations, varied athletic levels of players examined, and different foci of the FT exercises administered might account for these varied outcomes. Some tests (touch high, lane agility, lateral shuffle test, dribbling line drill, and free-throw) were used once among included studies, which might not be representative of the overall effectiveness of FT because there might not have been enough exposure or repetition, limiting the scope of evidence. Notably, some physical fitness attributes only received minimal attention (e.g., one to three studies investigating muscular endurance, balance, and flexibility), and no studies explored the effects of FT on body composition, reaction time, or coordination–all crucial aspects of basketball performance. Consequently, more research attention should be given to exploring the effects of FT on these attributes among basketball players moving forward. The content of the FT program directly influences training outcomes. Thus, practitioners should tailor the FT program according to the specific needs and skills of the basketball players they work with. A program length of more than 8 weeks may have a significant improvement in fitness and skill performance, whereby practitioners should carefully structure the FT stimuli to progress in difficulty and intensity over time.

## Data Availability

The original contributions presented in the study are included in the article/Supplementary material, further inquiries can be directed to the corresponding author.
